# Effects of Self-Myofascial Release on Athletes’ Physical Performance: A Systematic Review

**DOI:** 10.3390/jfmk9010020

**Published:** 2024-01-11

**Authors:** Luis Manuel Martínez-Aranda, Manuel Sanz-Matesanz, Ezequiel David García-Mantilla, Francisco Tomás González-Fernández

**Affiliations:** 1 Physical and Sports Performance Research Centre, Faculty of Sports Sciences, Pablo de Olavide University, 41013 Seville, Spain; 2 SEJ-680: Science-Based Training (SBT) Research Group, Faculty of Sports Sciences, Pablo de Olavide University, 41013 Seville, Spain; 3 Faculty of Sport, Catholic University of Murcia, Guadalupe, 30107 Murcia, Spain; msanz74@alu.ucam.edu (M.S.-M.); ezequiel.davidgarcia@gmail.com (E.D.G.-M.); 4 Department of Physical Education and Sports, Faculty of Education and Sport Sciences, Campus of Melilla, University of Granada, 52006 Melilla, Spain; ftgonzalez@ugr.es

**Keywords:** self-fascial massage, intervention programme, foam rolling, range of motion, athletes, fitness performance

## Abstract

Therapists and strength and conditioning specialists use self-myofascial release (SMR) as an intervention tool through foam rollers or massage rollers for soft tissue massage, with the purpose of improving mobility in the muscular fascia. Moreover, the use of SMR by professional and amateur athletes during warm-ups, cool downs, and workouts can have significant effects on their physical performance attributes, such as range of motion (ROM) and strength. The purpose of this study was to analyse the literature pertaining to these types of interventions and their effects found in different physical performance attributes for athletes. A systematic search was carried out using the following databases: PUBMED, ISI Web of Science, ScienceDirect, and Cochrane, including articles up to September 2023. A total of 25 articles with 517 athletes were studied in depth. SMR seems to have acute positive effects on flexibility and range of motion, without affecting muscle performance during maximal strength and power actions, but favouring recovery perception and decreasing delayed-onset muscle soreness. Some positive effects on agility and very short-range high-speed actions were identified, as well. In conclusion, although there is little evidence of its method of application due to the heterogeneity in that regard, according to our findings, SMR could be used as an intervention to improve athletes’ perceptual recovery parameters, in addition to flexibility and range of motion, without negatively affecting muscle performance.

## 1. Introduction

The myofascial system consists of a continuous three-dimensional, fibrous, soft connective collagen tissue, which envelopes the body [1]. This includes elements such as adipose tissue, neurovascular sheaths, aponeurosis, deep and superficial fascia, joint capsules, ligaments, membranes, meninges, myofascial expansions, periosteum, retinaculum, tendons, visceral fascia, and all intramuscular and intermuscular connective tissues, including the endomysium, perimysium, and epimysium [2]. The fascial system penetrates and surrounds all organs, muscles, bones, and nerve fibres; it gives the body a functional structure and provides an environment that allows all body systems to operate in an integrated manner [3]. The myofascial system and its physiological effects on the human body have been widely studied over the past decade within the field of physical activity and sports strength and conditioning [4,5,6]. Although scientific evidence is limited, nowadays, it is common to see the terms “myofascial release” (MFR) and “self-myofascial release” (SMR) in areas frequented by the general population with individuals of all ages and abilities (e.g., gyms or sports centres) [7,8], and even more in the fields of sports performance (athletes and their coaches) and physiotherapy [9,10].

SMR is a technique based on applying pressure to specific areas of the subject with their own bodyweight. This self-massage is performed by rolling on the floor with a foam roller (FR), which can be of different textures, sizes, and even vibrating characteristics. There are also other commonly used tools such as the roller massager (RM), lacrosse balls, Theraguns, and a therapeutic cane or “Theracane” [11,12]. Evidence suggests that these tools improve range of movement (ROM) [13] and recovery processes by decreasing the acute effects of delayed-onset muscle soreness (DOMS) on post-exercise muscle performance [14,15].

Despite its popularity, the physiological effects of many SMR tools on the body remain unclear. Consequently, a consensus on the specific utilisation of SMR in an optimal programme for enhancing physical capabilities, accelerating recovery processes, and improving overall athletic performance is yet to be established [16,17]. Considering that SMR has been developing as a trend in physical conditioning [18], it is important to identify the conceptual meaning of the myofascial system for a broader understanding of the effects of SMR on the human body and how these can affect athletes’ performance. Therefore, studies by authors such as Weerapong et al. [19] have divided the possible effects of this type of intervention on the human body into four categories: biomechanical, physiological, neurological, and psychological. Other authors have differentiated two more types of categories: mechanical and neurophysiological [20]. Mechanical mechanisms include SMR thixotropy [2], piezoelectricity [2,13], fascial adhesions [21,22], cellular responses [14,23], fluid flows [24,25], fascial inflammation [26,27], and myofascial trigger points [28,29]. However, many of these mechanical mechanism theories have faced criticism due to the argument that pressures beyond the typical physiological ranges of human tissues are required to induce deformations in most tissues [30]. Nevertheless, it is believed that studying these mechanisms can offer an approach to gaining a better understanding of the physiological effects that SMR may have on the human body. When delving deeper into these mechanisms, specifically within the framework of fascial adhesions, it is proposed that the various fascial layers, which typically glide relative to one another, will undergo changes that cause them to stick together [21,22]. It is believed that these fascial adhesions are released by moving the affected body area through a full range of motion under traction [21].

Concerning the fluid flow model, it has been reported that since the stiffness of the fascia is affected by the liquid content it sustains, SMR could increase the plasticity of the fascial tissue through temporary changes in the liquid content; since the fascia expels excess fluid after compression, this would allow an increase in range of motion (ROM), before the tissue is rehydrated [5,14]. It should be noted that the foam roller, as an SMR instrument, has been proposed as a particularly effective tool for the purpose of increasing ROM [26]. On the other hand, models involving the effects of fascia inflammation suggest that the muscle or fascia hardens as a result of inflammation [27,31], where SMR can reduce inflammation by increasing blood flow. It is not yet clear if the muscle or fascia can be pathologically altered in this way, but there are indications that SMR and manual therapy, in general, can affect blood flow by increasing the production of nitric oxide [32,33]. Furthermore, fascial inflammation may also be connected to the concept of myofascial trigger points (MTPs). It has been suggested that these points occur when motor end plates release an excess of acetylcholine, leading to the shortening of sarcomeres, disruption of cell membranes, and damage to the sarcoplasmic reticulum, ultimately resulting in inflammation [27,28,34]. However, the phenomenon of MTPs has come to be questioned in terms of its reliability due to a lack of clinical evidence [35,36].

Although mechanical mechanistic studies of the effects of SMR on the organism were the first to be proposed [2,37], we believe that it is also important to mention studies of neurophysiological mechanisms for the effects that SMR can have on the human body. In this regard, some research has shown that muscle massage causes the inhibition of the H reflex [38,39,40,41,42], which is an indirect measure of the excitation of alpha motor neurons. This phenomenon has also been attributed to the activation of mechanoreceptors, which are believed to inhibit the central nervous system during massage [42]. It is noteworthy that Bradbury-Squires et al. [43] showed a decrease in electromyographic activity (EMG) during the exercise called “body weight front lunges” after an SMR session, which could offer a possible explanation based on the H reflex inhibition.

According to studies that outline the possible effects of SMR on the body, there are several effects that can occur after physical exertion; fascial restrictions can be among them, causing an inhibition of normal muscle function [2,3,44]. This affects the musculoskeletal system and the physical conditions that arise from it, which are essential for sports performance [45], such as strength, speed, endurance, and flexibility [46].

Over time, several types of body massages have been developed to address the problem of fascial restrictions with the aim of improving muscle function, ROM, and other physical fitness variables. Starting from the principle that, at greater efficiency of movement, there is lower injury risk [23], some of these therapies have been commonly used by sports medicine clinicians, strength and fitness specialists, and athletes with the purpose of improving overall physical performance [14].

On this basis, we can find studies such as that by Mauntel et al. [15], who conducted a systematic review that evaluated the effectiveness of several myofascial therapies on ROM, muscle strength, and muscle activation. The authors evaluated 10 studies that found significant improvements in ROM but no significant change in muscle function after the interventions. Meanwhile, Schroder et al. [47] conducted a review evaluating the effectiveness of SMR using specifically a foam roller (FR) and a massage roller for pre-exercise and recovery purposes. Of the 9 included studies, the authors concluded that SMR has positive effects on ROM and delayed-onset muscle soreness (DOMS). Likewise, Cheatham et al. [10] concluded that the SMR showed significant benefits in ROM, with improvements in muscle performance before and after exercise, as well. However, most recently, Wiewelhove et al. [48] analysed the effects of SMR with FR, concluding that the effects of foam rolling on performance and recovery were rather minor and somewhat negligible, though they could be relevant in some cases (e.g., to increase sprint performance and flexibility or to reduce muscle pain sensation). The evidence appears to support the extensive use of foam rolling as a warm-up rather than a tool for recovery. Lastly, Konrad et al. [49] and Alonso-Calvete et al. [50], focusing on performance parameters in the general population (healthy individuals mainly), found no conclusive results of physical performance tests on the effectiveness of the use of the FR; its use was recommended acutely, and not in protocols lasting longer than 2 weeks.

Interestingly, there is no evidence of any systematic review that synthesises the effects of SMR specifically in athletes of different sport disciplines, and nor can there be found evidence of reviews studying the effects of SMR resulting from the use of other SMR tools besides the FR and RM. Therefore, this review aims to analyse the effects of SMR on several physical performance variables evaluated in athletes. As specified earlier, SMR techniques currently represent a deeply rooted and widely utilised method in the athletic population. However, there is a need for greater consensus and a more in-depth study of their application methodologies, the most commonly targeted areas, and the primary effects demonstrated on performance following their use. This information will enable the formulation of more precise recommendations for interventions of this nature, aiming to tailor those so that they could be considered optimal for enhancing athletic performance. Additionally, this information could offer a valuable resource to support athletes and coaches to implement new performance enhancement strategies or set aside ineffective protocols, making training more efficient.

## 2. Materials and Methods

### 2.1. Search Strategy

This systematic review was carried out in accordance with the recommendations of the Preferred Reporting Items for Systematic Reviews and Meta-Analyses (PRISMA) guidelines [51].

The studies were identified by searching the following electronic databases: MEDLINE (PubMed), Web of Science (WOS), ScienceDirect, and Cochrane. A time search parameter was established between 1 January 2008 and 1 September 2023, using the Patient, Intervention, Control, and Results (PICO) strategy [52], where an adequate construction of the research question and the review of the literature was required based on the following keywords: “athletes”, “sports”, “myofascial release”, “Self-Myofascial Release”, “Foam rolling”, “performance”, “delayed onset muscle soreness”, “DOMS”, “range of motion”, “flexibility”, “strength”, “muscle activation”, “power”, “force”, “agility”, and “sprint”.

The following Boolean operators were used following the PICO strategy for the methodological reliability of the search: (“Myofascial release” OR “Self-Myofascial Release” OR “foam rolling” OR “Self-fascial massage” OR “self-massage”) AND (performance OR “Sports performance” OR “fitness performance” OR “physical performance” OR “delayed onset muscle soreness” OR “effects on muscle” OR “Range of motion” OR flexibility OR strength OR “muscle activation” OR power OR force OR agility OR sprint) AND (athletes OR Sports).

The search strategy used in this study was adapted to the specific conditions of each database’s search engine. In this case, an advanced search approach was consistently employed, with identical filters for temporality and study type (original research).

### 2.2. Study Selection

The following inclusion and exclusion criteria were taken into consideration:

#### 2.2.1. Inclusion

(1) The subjects had to engage in sports practice for more than or equal to five hours a week. (2) The subjects had to have been involved in one or more competitive sports disciplines for at least six months or equivalent in training hours. (3) The studies had to measure the effect of SMR in one or more functional physical-sports performance-related factors. (4) The subjects had to use an SMR instrument to demonstrate the respective effects in each intervention. (5) Articles had to compare the effects of SMR/FR using two or more groups with different protocols (including a control group or at least one group without SMR/FR).

#### 2.2.2. Exclusion

(1) Studies in a language other than English. (2) Case reports, conferences, and systematic, literary, or narrative reviews with or without meta-analysis. (3) Studies concerning injury rehabilitation programmes or invasive interventions. (4) Studies involving pregnant women, cancer patients, or other pathologies. (5) Studies including injured athletes or a non-athlete population.

### 2.3. Quality Assessment

To evaluate the quality of the studies, the PEDro scale [53] was used based mainly on the independent consensus by the authors: LMMA and MSM. This tool allows one to quickly identify which of the randomised trials may have sufficient internal validity and statistical information to make its results interpretable. The scale is composed of 11 criteria, and one point is awarded for each criterion clearly met. According to the scale, after applying the inclusion and exclusion criteria, all the selected articles obtained a score of 6 or higher and were accepted in this review (Table 1).

### 2.4. Data Extraction and Synthesis

From the selected studies, the following data were extracted from each article: study objective, group of participants, type of intervention, methods, measurement, and main results or highlights. For the qualification of the results of each study, and in order to homogenise the findings as much as possible, the significance levels (*p*-value) are given in the results section for a more functional comparison. Information on the mean and standard deviation is also provided in the text, where possible.

To facilitate the reader’s understanding of the studies, the main results obtained are subdivided into categories related to the performance variables that each intervention protocol focused on. These variables include flexibility/mobility, strength, speed, agility, or the perception of effort and recovery.

### 2.5. Search Summary

**Figure 1:**
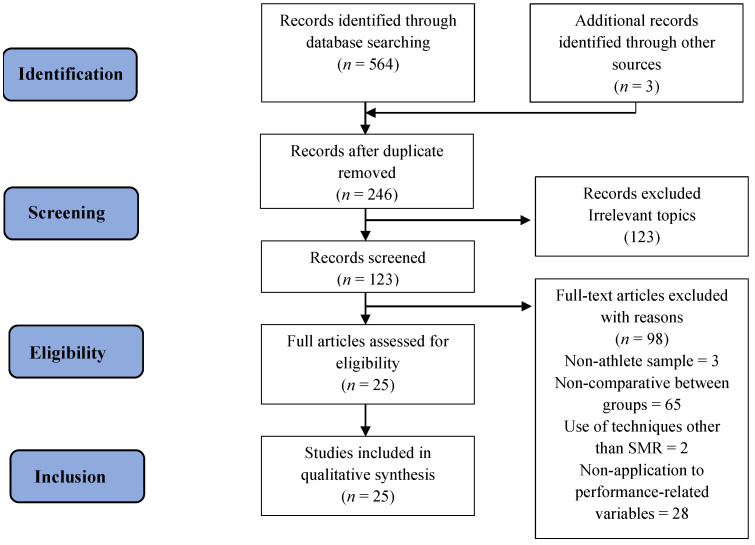
PRISMA flowchart for search methodology.

The PRISMA methodology was used, consisting of a list of 27 items [79] and a four-phase flow chart [80] (Figure 1). A total of 567 articles were initially identified through the databases and 3 additional records were found in other sources. After deleting the duplicate articles and carefully reading the abstracts, 246 articles were selected, of which 123 were chosen after reading the full text. Then, 98 articles were excluded for not meeting the inclusion/exclusion criteria. Finally, 25 studies were included in this systematic review.

## 3. Results

### 3.1. Characteristics of Included Studies

In the 25 studies that met the inclusion criteria for this review, a total of 517 athletes (363 men, 154 women) were counted, with an age range between 14 and 37 years. Due to the methodological diversity of each study included in this systematic review their general characteristics are listed in Table 2. This includes the type of design of each study, a brief description of the subjects specifying the number of people involved in each study, their respective genders, the sports experience counted in years and level of sports competition, the regions of the body intervened, and finally, a description of the interventions performed and the total number of sessions recorded.

### 3.2. Study Type

According to the study type, twelve were randomised controlled trials (RCTs) and the rest were randomised crossover designs (RCDs). The sample size was also taken into account, differentiating between the control group and experimental group and by sex and mean age of subjects, with thirteen studies working with a sample equal to or less than sixteen subjects. Most of the studies chose as the region of application areas of the lower limb and only two studies covered the upper body.

Regarding the measurement of the effects of SMR on the physical performance variables, tables were created charting the results of each of the respective measured variables.

### 3.3. Main Results

#### 3.3.1. Mobility Improvement

Table 3 lists the results obtained concerning the improvement of mobility and flexibility in the study sample. Of the 20 studies analysing variables related to flexibility, 13 of them found significant results after the application of the FR in the selected sample.

In the straight leg raise tests, mainly oriented toward subjects’ posterior chain mobility, improvements were found in the study of Oranchuk et al. [63] in their FR (+7.3%, *p* < 0.001) and FR + heat (+13.1%, *p* < 0.001) protocols. Similarly, Gillot et al. [65] found improvements in this test for both the 20 and 40 s intervention groups, with an improvement of +18.6% (*p* = 0.004) in the right leg for the 20 s group, as well as +8.2% (*p* = 0.002) in the right leg and +20.6% (*p* = 0.003) in the left leg for the 40 s group. Applying the same test, Richman et al. [69] demonstrated an increase of +6.1% after FR application (*p* < 0.05), which was very similar to the light walking + dynamic stretch group. In the same spirit, Sağiroğlu et al. [70] and Markovic [77] also demonstrated significant differences after SMR application in their samples of combat sports athletes (*p* = 0.029) and football players (*p* = 0.039), but in both cases with comparable improvements or even less advantageous compared to other strategies, such as aerobic running (isolated) or fascial abrasion techniques. In any case, the flexibility level seemed to be better in the short term (<10 min) when aerobic running was combined with SMR [70].

Furthermore, the application of the FR for improvement on dorsiflexion tests was similarly demonstrated in several published works. Among them was the study by Romero-Franco et al. [64], where significant differences were found in the FR intervention group, both in the first data collection after the protocol (*p* < 0.001) and at 10 min after the end of the protocol (*p* = 0.014). In addition, Aune et al. [66] found significant increases over the four-week duration of the study (*p* < 0.001); however, no significant differences between the two groups were found (*p* = 0.937). Finally, Škarabot et al. [76] demonstrated significant differences in both the SS and the FR groups, with the FR being more beneficial in the combined FR+ static stretching group, though they found *p* values < 0.05 in all cases.

Regarding specific hip mobility tests, FR has also demonstrated its efficacy. Romero-Franco et al. [64] showed positive results in hip extension tests after their FR intervention protocol, with significant differences in measurements just after the end of the protocol and 10 min after the protocol (*p* < 0.05), but despite the better results for the FR vs. control, no significant differences between groups were found. Likewise, Guillot et al. [65] demonstrated significant differences in specific hip extension tests, both in their 20 s protocol (right leg +9.8%, left leg +8.8%, *p* < 0.001) and in the 40 s protocol (right leg +8.7%, left leg +7.8%, *p* < 0.001). Within the same study, significant improvements were shown in the hip flexion test with active mobilisation of the flexed leg, with improvements in the 20 s (right leg +16.5%, *p* = 0.004; left leg +12.9%, *p* = 0.01) and 40 s (right leg +19.7%, left leg +18.9%, *p* < 0.001) groups. In the same spirit, Behara & Jacobson [74] demonstrated significant changes compared to baseline (*p* = 0.000) after the application of different strategies, showing improvements using the FR protocol (15.6%) but also better results when using dynamic stretching (19.9%) for the ROM of the hip flexion in a sample of first division American football players. Similar results to those were found by Chen et al. [56], where significant differences in knee flexion were observed between the group using the VFR (vibration foam roller) plus DS and the group undertaking a general warm-up, in a study of elite female handball players (79.4 DS + VFR vs. 69.3 general warm-up, *p* < 0.05). On the other hand, Murray et al. [75] found significant improvements in overall flexibility following the administration of an FR programme (*p* = 0.03), indicating that an applied force equivalent to 50 ± 12.6% of the body weight (27.2 kg) could be appropriate to achieve the best results. Moreover, the application of this type of strategy increased the temperature of the muscles involved (control being colder by 0.15–0.17 °C, *p* < 0.01).

Finally, the work conducted by Fairall et al. [72] was the only study focused on specific upper-limb mobility tests. In this research, despite not finding significant differences between protocols, it was observed that the application of FR programmes combined with SS produced greater increases in ROM (10.15° ± 4.95 improvement) than isolated stretching (8.6° ± 4.4 improvement) or FR protocols (3.8° ± 1.4 improvement) in baseball and softball players.

Contrary to the results so far provided, the study by Kurt et al. [54] demonstrated significant differences in elite female handball players, but in this case, they reported better results in the DS group compared to the FR group in a sit-and-reach test (SMR 36.9 vs. DS 38.3).

#### 3.3.2. Strength Improvement

The studies related to the correlation between SMR interventions and athletes’ strength are shown in Table 4.

A total of 7 of the 17 articles achieved positive results with myofascial release programmes in strength exercises, with all of them being linked to jumping actions and one of them adding RM measurements in the upper body.

In the case of the CMJ test, which was the most commonly used by researchers, five articles found significant differences in the sample after the application of myofascial therapy. Romero-Franco et al. [64] reported significant differences in the intervention group both in the first data collection (+4 cm, *p* < 0.001) and 10 min after the protocol (+1.7 cm, *p* < 0.01), finding in the control group, which performed the same warm-up but without the incorporation of the FR, differences only in the first data collection, which were lower than in the experimental group (+1.9 cm, *p* < 0.05). No significant differences at the second data collection at 10 min (+1.5 cm, *p* > 0.05) were found for the control group. Moreover, Richman et al. [69] demonstrated the efficacy of the inclusion of a myofascial release programme combined with dynamic stretching, which improved the results of a CMJ test (+2.63 ± 3.74 cm, *p* = 0.021) and SJ (+1.72 ± 2.47 cm, *p* = 0.022) in a sample of 14 female volleyball and basketball players. In a similar vein, Kurt et al. [54] demonstrated improvements in the use of SMR compared to static stretches in professional female handball players (SS-SMR: −1.47 ± 0.43, *p* = 0.002), though they also showed that DS achieved slightly better results than an FR (DS-SMR: +1.21 ± 0.53, *p* = 0.03). On the other hand, Giovanelli et al. [67] reported significant results for the rate of force development (RFD) extracted from a CMJ test when performed three hours after an SMR session, which increased the force exerted by 38.9% (*p* = 0.024). Finally, Wang et al. [59], using a sample of high-level tennis players (*n* = 27), found the highest significant differences between the SMR application group and the control group (no intervention) at 7 min in different muscle areas (SMR 53.18 vs. control 47.92, *p* = 0.03).

Furthermore, two studies analysed the changes in a vertical jump test (sergeant jump) after the inclusion of myofascial release. Stroiney et al. [68] found significant differences in a group of 49 athletes in a vertical jump test after the application of a myofascial self-release programme (+2.54 ± 3.2 cm, *p* = 0.04), reflecting a greater increase than a group performing an instrument-assisted soft-tissue mobilisation protocol, though those did not reach significant differences. On the other hand, Peacock et al. [78] found significant differences in a sample of 11 NCAA tournament athletes (Division I and Division II) from different disciplines (*p* = 0.012), finding no differences in the group that performed mobility work without the inclusion of an FR.

In addition to the studies cited above, Peacock et al. [78] showed changes for the vertical jump test (*p* = 0.012), horizontal jump (*p* = 0.007), and in the indirect measurement of RM in a bench press (*p* = 0.024) in their sample of NCAA DI and II athletes. In this context, Kurt et al. [54] revealed significant differences in isokinetic tests applied to professional handball players’ right leg, both in flexion and extension, between the SMR group and the static stretching group (flexion *p* = 0.006; extension *p* = 0.038), despite not finding differences with the dynamic stretching group. Finally, Chen et al. [56] found differences in a stiffness test measured with a myometer in a sample of professional handball players between the FR group and a general warm-up group based on running, with greater improvements in muscle tone in the group not using an FR (general 292.89 vs. FR 253.33 N·m^−1^).

#### 3.3.3. Speed Improvement

Only two studies were found that showed significant differences in speed tests after the application of myofascial release programmes compared to the nine total studies that included such tests.

In Table 5, it can be seen that the study of D’Amico [71] reported a statistically significant difference, observing a decrease in running time for 800 m flat in a comparison between an SMR session and active recovery. Meanwhile, Peacock et al. [78] reported a statistically significant difference in the 37 m sprint test, also known as the “40-yard dash” (*p* = 0.002), when comparing the results between a dynamic warm-up and an SMR session prior to the test. It should be clarified that the 37 m sprint is a test where the subject performs a race at maximum speed in a straight line within an assigned distance; the duration of this distance is taken from the start line until the end line is reached.

#### 3.3.4. Agility Improvement

Regarding agility test results, four out of seven studies that included specific tests showed significant differences after the application of myofascial release programmes (Table 6).

Firstly, Chen et al. [60] demonstrated significant differences in a hexagon test in a sample of 15 elite taekwondo athletes after the application of myofascial release in combination with warm-up (*p* = 0.03), whereas these differences were not achieved with warm-up in isolation. These results are in line with those found by Wang et al. [59], who applied the same test to tennis players; after implementing an SMR protocol, improvements were found in the intervention group that were superior to those in the control group.

Moreover, the study conducted by Rey et al. [73], applied to professional football players (*n* = 18), revealed a statistically significant difference for the variable “time” when a *t*-test was performed (*p* = 0.028) that compared the control group and the experimental group (with an SMR session before the test).

Lastly, Peacock et al. [78] showed a statistically significant difference (*p* = 0.001) in the pro-agility test of 18.3 m (providing information on different athletic abilities, such as speed, change in direction, as well as acceleration and deceleration), evidencing an improvement in time in favour of the SMR group compared to a dynamic warm-up group, when applied to 11 athletes from NCAA DI and II.

#### 3.3.5. Influence on Subjects’ Recovery Capacity

The results shown in Table 7 list the differences found in the recovery test and perceived exertion of the subjects analysed, with only three studies finding significant differences among the eight studies included.

Rey et al. [73] showed significant differences in the total quality of recovery test (TQR) and visual analogue scale when applied after the training session, with significantly better results for the FR group (12.67 vs. 15.00, *p* = 0.018; and 4.83 vs. 5.6, *p* = 0.045, respectively).

Similarly, better results concerning recovery were reported by Rahimi et al. [62], showing lower scores in the FR group for the Hooper questionnaire (HI), especially on the second (*p* = 0.01) and third match days (*p* = 0.005) (post-recovery, 15 min after recovery, 180 min after the match, and at the end of the day). In addition, similarly better results for the FR were reported in terms of blood lactate on the third match day (*p* = 0.03) (post-recovery and 15 min after recovery).

Finally, Michalski et al. [58] reported significant positive differences in %MVC (GM and BF), especially right after applying the treatment, in favour of the HR group compared to the control. Similar findings were reported concerning sEMG values for GM, which were better for the HR group right after the rolling treatment, adding the follow-up in the case of the BF.

It should be noted that the studies by Lopez-Samanes et al. [61], Barrenetxea-García et al. [55], and Kozlenia & Domaradzki [57] did not find significant changes in any of the proposed tests after the application of the foam roller, so they have not been included in any of the textual descriptions within the categories analysed above.

## 4. Discussion

Given the wide use of SMR methods in the field of sports performance, this systematic review aimed to analyse the effects of SMR on several physical performance variables, which were evaluated in athletes.

In previous studies concerning the possible positive outcomes of using SMR, Schroeder and Best [47] stated that the results of FR use as a pre-exercise intervention or as a recovery strategy were neither homogeneous nor evident. In line with that, McKenney et al. [81] including 10 studies concerning orthopaedic conditions, and concluded that these studies produced few concrete conclusions from which truly useful practical applications could be established. Those authors suggested the need for more randomised controlled tests. Delving deeper into this topic, Beardsley and Skarabot [12] showed conflicting results related to the effects of the FR on flexibility, force development, sports performance, and the lag of DOMS.

Given that it has been a long time since such literature reviews were published, and that they were focused on other populations, the present systematic review intended to provide a comprehensive review of the effects of SMR interventions on certain measured variables of physical performance in athletes, such as flexibility, mobility, strength, speed, agility, and several factors involved in recovery. The findings of this systematic review can be considered important due to the wide use of SMR methods in the field of sports performance.

In this systematic review, 25 studies in total were identified [54,55,56,57,58,59,60,61,62,63,64,65,66,67,68,69,70,71,72,73,74,75,76,77,78], examining the use of different SMR techniques before and after exercise and also as a recovery method. Each study applied different exercise protocols, using various treatments, application times, and measures and assessing different results, which made the selection of results a challenge. Furthermore, due to that heterogeneity in the studies, it was difficult to conclude the correct form of use of SMR in the field of physical-sports training. However, by grouping the results of each study, some important findings were obtained.

Concerning mobility improvement and flexibility, SMR exercises can temporarily increase the ROM of the hip, knee, and ankle joints, as well as muscle flexibility, without affecting neuromuscular activity or maximum isometric strength in a non-athlete population [10,82,83,84,85], or recreational participants in running activities [6]. Specifically in an athlete sample, thirteen studies were found reporting an increased ROM when using SMR, mostly in the lower limbs [54,63,75], with only one in the upper limbs [72]. Meanwhile, several studies did not show significant changes or improvements when compared to conventional stretching (passive/static or dynamic) or other methods combined, but they did not find a negative effect at least [59,60,61,71,73,78]. In addition, other studies [86] reported a lack of consistency or evidence to support myofascial decompression as a functional tool to increase hamstring flexibility; or found that proprioceptive neuromuscular facilitation stretching induced more gains in hamstring flexibility compared to the FR/SMR [87].

Overall, although the underlying reasons for the improved flexibility remain uncertain in some ways, from a structural point of view, the positive effects observed may be explained by a temporary reduction in the connection between the fascial tissue and muscle tissue [7,88,89,90,91,92], or by plasticity deformation of the connective tissue. (e.g., fascia, tendon, capsule). From a functional perspective, a temporary reduction in pain perception may also lead to an improvement in the short-term flexibility [7,12,93,94,95]. Possibly for that reason, studies focusing on the short-term effects of the SMR report that knee and hip flexibility mainly improve immediately after treatment, while no evident positive effects are found after 24 h later [77]. Actually, according to short-term interventions, in line with the aforementioned temporal benefits, the effects on flexibility last less than 10 min.

Moreover, in some cases, the combination of SMR with static stretching has superior effects in increasing the ROM compared to one of these exercises performed in isolation [72,76]. Reinforcing this idea, other studies stated that the use of dynamic stretching or DS + FR [54,56,74,96], as well as aerobic running/combined aerobic running + SMR [70], improve sit-and-reach performance, knee flexibility, and hip ROM results to a greater degree compared to isolated FR/SMR, especially in the short term (<10 min). However, Warneke et al. [97] reported that any immediate enhancements in range of motion (ROM) could not be solely ascribed to foam rolling, conjecturing that warm-up effects might be accountable independently of the FR or replicating the rolling motion.

Consequently, it seems that in recommending the application of SMR exercises in athletes focusing on mobility and flexibility, that recommendation should be focused on improving the simple effects induced by traditional or simple stretching. In this case, it is important to apply enough pressure on the muscles, for a minimum period of 30–40 s or >60 s, and the application is much better when in combination with other methods such as dynamic stretching or moderate running.

Regarding the effects of SMR on strength performance, positive and negative results were found. Seven studies focused on athletes found statistically significant results in relation to SMR sessions prior to testing the squat jump, CMJ, drop jump, and vertical jump [54,59,64,67,68,69,78]. Additionally, Peacock et al. [78] showed an improvement in the long jump and the 1RM test on a bench press [78], leading them to recommend the use of SMR exercises with an FR to improve power and strength, especially when combined with a dynamic warm-up.

The enhancement in performance could stem from SMR serving as a standalone warm-up. It has been theorised that SMR may elevate skin temperatures and boost blood circulation to the muscle tissue [98]. This rise in blood flow and muscle tissue warmth might alleviate muscle restrictions and enhance range of motion (ROM) without impeding neuromuscular force production [82].

However, despite the above studies showed significant results, there are also studies reporting no significant improvements for interventions of SMR in terms of maximum force or power testing [12,57,60,61,62,66,70,73]. There have been no effects on several variables, such as vertical jump (height, power, and/or speed), 90°/s isokinetic knee extension, contraction time, and isometric force after SMR exercises [74,75,99,100].

In addition, aside from these studies without differences, a recent study stated that a general warm-up based on running yielded better results in muscle tension tests compared to those individuals using an FR, indicating a potential decrease in muscle tone and hamstring stiffness and, consequently, a reduction in the capacity for specific force application in elite female handball players [56].

It is true that a diminished sensation of muscular fatigue might enable individuals to prolong the duration and intensity of a single exercise session, potentially resulting in long-term improvements in performance [100]. However, based on evidence in the scientific literature, it seems that foam rolling does not have clear and evident positive effects on this kind of performance variable.

Therefore, due to the diversity of effects corresponding to SMR and strength–power parameters, a specific recommendation for use in athletes cannot be given. The same applies to any type of population, as indicated by similar previous findings focused on a healthy adult population [7,50].

Focusing on the effects of SMR on athletes’ speed, only two out of nine studies showed statistically significant results. In the study by D’Amico [71], the results of two sets of 800 m runners were taken. One group performed a warm-up followed directly by the 800 m, and the other group performed a warm-up followed by SMR exercises in the lower body. When contrasting the two groups, an improvement was observed in race time for the group performing a warm-up followed by SMR exercises (145.2 ± 1.8 vs. 146.9 ± 2.2 s; difference of 1.7 ± 0.4 s). Moreover, for a shorter distance, Peacock et al. [78] analysed a 37 m race test at the maximum speed, where a shorter run time was obtained in the group that performed a pre-race SMR session (4.95 ± 0.21 s) compared to a dynamic warm-up before the race (5.11 ± 0.29 s).

Nevertheless, it is important to highlight the heterogeneity in the studies analysing the effects of SMR on speed; despite to carrying out the same protocols/speed tests, the measuring instruments were different and this could indeed have been a differentiator in the results. One good example of this is the study by Stroiney [68], which showed a lack of significant results in the same 37 m race test, and where instead of using a foam roller, a massager roller was the instrument chosen. This implies another type of pressure on the fascia and muscles, since the subject holds the roller with their hands and applies the desired pressure, in comparison to the foam roller, where the subject generates the pressure through their bodyweight on the applied area. However, other previous studies reported no significant differences between massage types and sprint/speed performance [55,59,61,62,73,99,100,101,102].

In this regard, and when trying to find possible reasons for the lack of positive results when using SMR in relation to strength (especially vertical jump) or speed parameters, prior research has indicated that different massage modalities can reduce elastic storage and neural drive and enhance parasympathetic activity [103]. Several advantages can come from a more compliant muscle, but in activities requiring power, heightened compliance might lead to reduced elasticity and force transfer capacity [101]. The relaxation response, evaluated through the H-reflex, has been a subject of study, with certain investigations noting a decline in H-reflex amplitude post-massage. Changes in neuromuscular inhibition and a lower alpha motor neuron excitability have been found [41], which are related to those muscle groups undergoing massage and potentially occur at the mechanoreceptor level. This could be one of the potential explanations for the absence of positive results after the use of SMR, especially considering that it is applied primarily to the musculature involved in the assessed action/test.

In light of these findings and conclusions, it is indeed challenging to recommend SMR strategies as a tool for improving speed in athletes.

Interestingly, other investigations studied variables indicating the effects of SMR on agility. Of the twenty-five investigations analysed, only seven performed tests to study SMR and agility, where four studies found significant results [59,60,73,78]. In the agility “*t*-Test” and “Hexagon Test”, significant results were obtained in time performance [59,60,73]. Moreover, Peacock [78] reported significant positive results in the performance time for the “Pro-Agility 18.3 m” test, specifically when an SMR session was compared with a dynamic warm-up session prior to the test.

As a consequence, although these results are related to agility, the physical capacity that predominates in those tests is the maximum speed over very short distances that athletes achieve, which could complement some of the aforementioned positive effects of SMR on speed. Therefore, although there is very little evidence, these results support that SMR exercises prior to very short-range and high-speed actions implying accelerations and decelerations may have positive results for athletes; however, a larger number of studies are needed to prove and reinforce this conclusion [50].

Beyond this, of the twenty-five studies compiled in this systematic review, eight s studied the effects of SMR on factors that influence athletes’ recovery. The factors that these studies covered were rate of perceived exertion (RPE) [55,60,62,67], recovery perception [73], the level of tiredness [62,63], blood lactate [62,71], and parameters related to muscle contraction and electrical potential [58,75].

In this regard, the analysed studies showed that there were no significant changes in the rate of perceived exertion in athletes between the pre-tests and the post-tests, where SMR exercises were related to strength tests, such as CMJ and the squat jump, or speed tests [55,60,62,67].

Concerning recovery perception and tiredness, Rey et al. [73] showed significant data relating SMR and perceived recovery using the TQR test as a measurement tool, complemented by information derived through a similar measure of the VAS fatigue. These two tests were compared with passive recovery. The study revealed that athletes who performed SMR exercises after football training sessions had better perceived recovery and less feeling of tiredness 24 h after the exertion during training, compared to those athletes performing a passive recovery. This statement has been supported by other studies of the athlete population, which applied co-pressure methodologies in potentiation and recovery, finding positive results in the reduction in delayed pain [104]. In the same spirit, Rahimi et al. [72] reported better recovery results (lower HI scores) for the FR group compared to a passive rest group, especially on the second and third match days. In addition, a better blood lactate clearance was reported for the FR group during that period.

Moreover, it seems that utilising SMR on the hamstring muscles induces alterations in the electrical potential of the muscles in the lower limbs. These alterations take place in structures that undergo SMR, and interestingly, also in very close muscles, where SMR is not applied [58]

Those studies show that SMR has beneficial effects on some of the variables related to recovery. But despite presenting positive results in reducing the fatigue sensation and changing the electrical potential in muscles after training or competition, the general findings are not strong enough to recommend SMR as an effective tool to improve recovery in athletes in a decisive way.

Finally, we must note that this study has not been free of limitations and difficulties. Firstly, the majority of the studies we reviewed were focused on the lower limbs, compared to limited research on the potential benefits of SMR techniques in the upper limbs, with only one study found in this regard. The muscular demands in various sports reliant on the upper limb musculature are evident, and the prevalence of pathologies in this area is significant, particularly in the shoulder [105] and cervical [106] regions. Therefore, studying diagnosis and intervention in this context appears to be fundamental.

As an additional limitation, the sample size was rather low. Therefore, the possibility of specifying SMR programmes for specific sports is limited. This is an important factor to consider, as athletes have particular physiological, anatomical, psychological, and social characteristics depending on the sport discipline in which they are involved. Additionally, because most studies reviewed used a foam roller, a limitation in understanding the effects that other SMR instruments may have on the factors involved in sports performance has also been identified.

Lastly, it seems that the vast majority of potential side effects of SMR exercises have been ignored or not studied in the scientific literature.

## 5. Conclusions

After analysing articles that studied the effects of SMR on factors related to physical performance in athletes, it can be stated that SMR exercises can serve as effective alternatives to improve the flexibility in the ROM of athletes’ joints, both when applied in isolation (in a lesser extent) and when combined with static and especially dynamic stretching. In addition, this gain can be achieved without negatively affecting muscle activity or performance manifested in strength, speed, and agility. The improvements in flexibility allow for greater performance in movement patterns and consequently decrease the risk of skeletal muscle injuries. Some of these improvements in the ROM of athletes were observed during applications that lasted between 30 s and 15 min; however, the most common use was found for around 1 min 30 s in the muscle area.

It has also been evidenced that SMR can have beneficial effects as an instrument of recovery by increasing the quality of recovery perception and reducing the pain perception, fatigue, and delayed-onset muscle soreness. In addition, it can improve the electrical potential response of the muscles where SMR is applied, including nearby muscles involved in the movement to be analysed.

In any case, the application of myofascial release methods is a widely used tool by athletes from different disciplines nowadays. However, due to the heterogeneity of the methods applied between each study, it is not possible to reach an ideal consensus on an SMR programme for athletes. Furthermore, there is a lack of complete certainty about its positive effects on certain sports performance aspects (e.g., strength-related parameters or general speed performance).

These issues represent the main gap in research focused on this topic. Based on this, it can be concluded that SMR application is positively associated with enhancing tissue flexibility, ROM, and perceptual factors, and so it should be considered by athletes and coaches in their routines focused on these capacities. However, the application of SMR techniques aimed at other objectives, such as improving strength, power, and overall speed, currently lacks real certainty, meaning such methods may be dispensable, thereby increasing the time efficiency of training sessions.

Certainly, based on current scientific evidence and the small number of studies with positive results related to the effects of SMR on some physical performance factors in athletes, SMR exercises should be used and approached with caution, applied only for certain objectives, and with consideration the variables for which they have clearer benefits.

## 6. Brief Practical Applications

Based on recent research, the combination of SMR exercises with a dynamic warm-up, as well as dynamic and/or static stretching, could be used for improved functionality. In addition, the combination of SMR exercises with specific low loads of muscle activation may come close to obtaining an ideal warm-up protocol for different sports, whether they are performed in group or individually. Although the ideal duration and pressure of the SMR exercises are uncertain, it seems that prolonged durations, of around 2 min, with greater pressure exerted on the body, could lead to greater improvement.

Therefore, it can be theorised that SMR applications throughout the body, using dense foam rollers, massage rollers, and lacrosse balls for controlled periods of time, can be effective in some ways. They may offer benefits in enhancing simple/short manifestations of explosive performance (agility and very short/high-speed actions), flexibility in some muscle structures, and especially ROM, while preserving strength and power. Furthermore, SMR applications may be interesting for alleviating, reducing, and/or improving certain perceptual factors, such DOMS, fatigue, and pain, resulting from resistance training.

Finally, engaging in light exercise as a form of active recovery is probably a more effective approach than an FR in minimising performance declines induced by fatigue during subsequent exercise sessions.

## Figures and Tables

**Table 1:** PEDro scale for study qualification.

Studies	1	2	3	4	5	6	7	8	9	10	11	S
Kurt, 2023 [54]	1	1	0	1	0	0	0	1	1	1	1	7
Barrenetxea-García, 2023 [55]	1	1	1	1	1	0	0	1	1	1	1	8
Chen, 2023 [56]	1	1	0	1	0	0	0	1	1	1	1	7
Kozlenia 2022 [57]	1	1	0	1	0	0	0	1	1	1	1	7
Michalski, 2022 [58]	0	1	0	1	0	0	0	1	1	1	1	6
Wang, 2022 [59]	1	1	0	1	0	0	0	1	1	1	1	7
Chen, 2021 [60]	1	1	0	1	0	0	0	1	1	1	1	7
Lopez-Samanes, 2021 [61]	1	1	0	1	0	0	0	1	1	1	1	7
Rahimi, 2020 [62]	1	1	0	1	0	0	0	1	1	1	1	7
Ornachuk, 2019 [63]	1	1	0	1	0	0	0	1	1	1	1	7
Romero-Franco, 2019 [64]	1	1	0	1	1	0	1	1	1	1	1	9
Guillot, 2019 [65]	1	1	0	1	0	0	0	1	1	1	1	7
Aune, 2018 [66]	1	1	0	1	0	0	0	1	1	1	1	7
Giovanelli, 2018 [67]	1	1	0	1	0	0	0	1	1	1	1	7
Stroiney, 2018 [68]	1	1	0	1	0	0	0	1	1	1	1	7
Richman, 2018 [69]	1	1	1	1	0	0	0	1	1	1	1	8
Sağiroğlu, 2017 [70]	0	1	0	1	0	0	0	1	1	1	1	6
D’Amico, 2017 [71]	1	1	0	0	1	0	0	1	1	1	1	7
Fairall, 2017 [72]	1	1	1	1	0	0	0	1	1	1	1	8
Rey, 2017 [73]	1	1	1	1	1	0	0	1	1	1	1	9
Behara, 2017 [74]	1	1	1	1	1	0	0	1	1	1	1	9
Murray, 2016 [75]	1	1	0	1	1	0	0	1	1	1	1	8
Škarabot, 2015 [76]	1	1	1	1	1	0	0	1	1	1	1	9
Markovic, 2015 [77]	0	1	1	1	1	0	0	1	1	1	1	8
Peacock, 2014 [78]	0	1	1	0	0	0	0	1	1	1	1	6

S: score; 1. The selection criteria were specified; 2. The subjects were randomised to the groups (in a cross-study, the subjects were randomly distributed as they received the treatments); 3. The assignment was hidden; 4. The groups were similar at the beginning in relation to the most important prognostic indicators; 5. All subjects were blinded; 6. All therapists who administered the therapy were blinded; 7. All evaluators who measured at least one key result were blinded; 8. The measurements of at least one of the key results were obtained from more than 85% of the subjects; 9. Initially assigned to the groups, and results were presented from all subjects who received treatment or were assigned to the control group, or when this could not be achieved, the data for at least one key result were analysed for “intention to treat”; 10. The results of statistical comparisons between groups were reported for at least one key result; 11. The study provides specific measures and variability for at least one key result.

**Table 2:** General characteristics of the studies that used SMR on athletes.

					Intervention	
Study (1st Author and Year)	Design	Subjects (Age)	Sports Experience	Muscle Groups	Duration	N° Sessions
Kurt, 2023 [54]	RCD	23 w (21.8 ± 1.73)	Turkey Women’s Handball Super League and regional league (9.57 ± 2.54 years of experience)	Quadriceps, hamstrings, hips	Three warm-up protocols in a randomised order: SS, DS, SMR All protocols start with 5 min cycling SS: 9 min of SS, 3 exercises 3 × 30 s both sides DS: 9 min of DS, 8 exercises 2 × 20 s both sides SMR: 9 min of SMP, 3 × 30 s each muscle group both sides	3 (1 per condition)
Barrenetxea-García, 2023 [55]	RCT	14 m and 16 w (20 ± 3.84)	Male First Regional League and Female Second National league in Waterpolo (more than 9 years of experience)	Gluteus medius, tensor fasciae latae, adductor, lumbar region, upper back, back of the shoulder and pectoral	FRG: 1 set of 60 s for each muscle group, 10 min in total (7-weeks, 28 sessions) CG: no intervention (passive, not using FR technique)	28
Chen, 2023 [56]	RCD	10 w (21 ± 1)	Taiwanese handball collegiate national champion team (Training more than 15 h per week)	Quadriceps and hamstrings	Three warm-up protocols in a randomized order: GW, DS and DS + VR All protocols start with 5 min jogging GW continues with 8 min of SS and 8 min of DS DS continues with 4 sets of exercises (8 min) DS + VR continues with 4 sets of DS exercises (8 min) and 4 sets of VR—30 s at a rate of 30 rolls per min (1 s up, 1 s down) in each muscle for both legs	3 (1 per condition)
Kozlenia 2022 [57]	RCD	14 m and 16 w (21.8 ± 1.15)	University amateur athletes (soccer, handball, basketball, volleyball and extreme conditioning program training) (3.76 ± 1.73 training sessions per week; 104.83 ± 26.01 single training session duration in min; 6.48 ± 3.12 weekly training volume in hours/week)	Calves, hamstrings, glutes, and thighs	Group A: 10 min GW (5 min jogging, 15 reps—air squats. 15 reps—high knees, 15 reps—lunges, and submaximal trials of the jump to be tested) + SI-SMR Group B: Only GW SI-SMR protocol: Each muscle group treated for 15 s with an intensity of 20 reps/15 s × leg, maintaining high pressure on the foam roller during application, 7–8 on the pain numbering rating scale	2 (1 per condition)
Michalski, 2022 [58]	RCT	40 m (A = 20 m, 25.5 ± 5.2; B = 20 m, 26.3 ± 1.3) (17 FP and 3 GK)	Soccer players of the regional soccer league; high level of physical fitness (soccer training a minimum of 3 times per week)	Hamstring muscles (FR application), assessing biceps femoris and gluteus maximus	Group A = hamstring group; protocol of 210 s applying FR technique; application rhythm per repetition: 4 s (2 s in one direction and 2 s in the opposite one) Group B = rest	1
Wang, 2022 [59]	RCD	27 m (20.4 ± 1.3)	Tennis players; 10 ± 0.7 years of experience	Lower back, gluteus, quadriceps, lateral thigh muscles, hamstrings, calf muscle groups	VFR = 5 min jogging + 1 set × 30 s (40 beats per min) each muscle group (7 min in total); PVPD = 5 min jogging + PVPD 1 set × 30 s each muscle group (7 min in total); CG = 5 min jogging	Only 1, (every subject performed only 1 condition)
Chen, 2021 [60]	RCD	15 m (20.63 ± 1.18)	Elite taekwondo athletes; 9.79 ± 2.77 years of experience	Quadriceps and hamstrings	Three warm-up protocols in a randomised order: GW, GW + VR, and GW with double VR for the weaker leg Three (GW + VR) or six sets (GW + double VR) × 30 s at a rate of 30 rolls per min (1 s up, 1 s down) in each muscle for both legs	3 (1 per condition)
Lopez Samanes, 2021 [61]	RCD	11 m (20.64 ± 3.56)	High-performance tennis players (ATP players among 300 best national tennis players in Spain)	Quadriceps, hamstrings, gluteus, gastrocnemius	Rolling for 8 min on each lower extremity unilaterally (two different warm-up protocols: dynamic vs. Self-Myofascial Release with foam rolling)	2 (1 per condition)
Rahimi, 2020 [62]	RCT	16 m (19.1 ± 1.3)	Elite futsal players (training hours per week = 9)	Anterior thigh, hamstrings, gluteus, and gastrocnemius	Five reps × 40 s with 20 s rest between repetitions (two groups: (i) passive recovery (PR); and (ii) FR recovery)	Three matches in five days
Oranchuk, 2019 [63]	RCD	26 w (19.4 ± 1.7)	NCAA Division II lacrosse (13) and soccer players (13) Years of experience: 6.9 ± 4.1	Hamstrings	SH, FR, SH + FR in combination, and control groups taken into consideration Three sets × 1 min with 30 s passive rest between sets (FR protocol)	4 (1 per condition)
Romero-Franco, 2019 [64]	RCT	18 m and 12 w (24.1 ± 4.2)	Collegiate competitive athletes (several disciplines)	Anterior thigh, hamstrings, calf	Experimental (8 min jogging and FR exercises) and control group (8 min jogging) For 45 s in each muscle of both legs + 15 s of rest between legs (the entire FR protocol lasted about 6 min)	1
Guillot, 2019 [65]	RCT	30 m (18.85 ± 1.10);	Professional rugby players	Right and left sides, separately (hip extensors, hip adductors, knee extensors, knee flexors and plantar flexors)	Participants from the FR 20 s and FR 40 s groups, respectively, performed a 7-week (15-session) foam rolling training programme involving between 7 and 14 back-and-forth movements per session; each back-and-forth movement did not exceed 3 s. CG with neutral task (cycling)	15
Aune, 2018 [66]	RCT	23 (18 ± 1). (11 w; 12 m)	Top-division Norwegian soccer club	Gastrocnemius	Participants were allocated to an FR or eccentric exercise intervention group, both designed to improve dorsiflexion ROM. Three bouts of 60 s of foam rolling. Bouts were separated by 30 s. Participants completed three separate testing sessions on day 1 (baseline and 30 min post), day 2 (24 h post), and day 28 (4 weeks post)	3
Giovanelli, 2018 [67]	RCD	13 m (26.3 ± 5.3)	Soccer, track and field, trail running, parkour; hours per week: 9.9 ± 3.5	Plantar fascia, gastrocnemius, tibialis anterior, anterior thigh with extended knee, anterior thigh with flexed knee, posterior thigh, gluteus, fasciae latae	SMR protocol in experimental condition, while in the control condition testing session, the same measurements are performed without undergoing the SMR protocol Protocol: 1 min × muscle group, with 10 s per change; The application frequency was about 0.5 Hz (e.g., each rolling cycle lasted about 2 s)	2 (1 per condition)
Stroiney, 2018 [68]	RCT	49 (20.35 ± 2.56). (21 w), (28 m).	Various sports; average days per week: 5.13 ± 1.16; average minutes per session: 83.34 ± 34.60	Sural triceps, hamstrings, quadriceps	SMR and IASTM groups SMR protocol: 90 s × muscle group	1 (each subject performed only one condition)
Richman, 2018 [69]	RCD	14 w (19.8 ± 1.3)	NCAA DII Volleyball and Basketball	Hip flexors, quadriceps, adductors, fascia lata tensors, plantar flexors and extensors	6-min per session. Group 1: Light walking; Group 2: SMR 30 s × muscle group × leg	2 (1 per condition)
Sağiroğlu, 2017 [70]	RCD	16 m (23.9 ± 3.6)	MMA (Judo, Karate, Tae Kwon Do, Muay Thai); years of experience: 12.9 ± 5.2	Hamstrings, quadriceps, gastrocnemius, soleus, glutes	SS and SMR groups SS = 4 stretching exercises; two sets × 30 s with 10 s passive recovery on each extremity; SMR = five back-and-forth FR movements × 30 s pressure with highest tolerable level. Two sets × 20 s with 10 s passive recovery × muscle × leg. Rest for 30 s between exercises	3 (1 per condition)
D’Amico, 2017 [71]	RCD	16 m (20.5 ± 0.5)	Track, 800 m flat	Glutes, hip flexors, quadriceps, iliotibial bands, adductors	The subjects complete two 800 m runs on a treadmill, separated by a 30 min rest, during which time a foam rolling protocol or passive rest period is performed FR protocol: Six back-and-forth FR movements on each side, adding up to a total of 30 s since each rolling movement lasts on whichever side for 5 s; 10 min per session	2 (1 per condition)
Fairall, 2017 [72]	RCD	12 m (36.92 ± 11.17)	Amateur baseball and softball; years of playing time: 28.42 ± 10.93	Infraspinatus and deltoids	SMR, SS, and the combination of both (SMR + SS) Protocol: Two sets of SMR × 60 s, 30 s rest per set; SS for 30 s × three sets + 30 s rest between sets; and combining SMR and SS	3 (1 per condition)
Rey, 2017 [73]	RCT	18 m (26.6 ± 3.3)	Professional soccer players; years of systematic soccer training: 14.8 ± 2.6	Quadriceps, hamstrings, adductors, glutes, gastrocnemius	FR recovery group and passive recovery group Protocol: 3 min after training session. Five FR exercises at a 50-pulse cadence per min. Both legs for 45 s each, with 15 s rest. The total FR time is 20 min	1 (each subject performed only one condition)
Behara, 2017 [74]	RCT	14 m (≥18)	NCAA DI American Football with >6 years of experience	Hamstrings, quadriceps, gluteus maximus, gastrocnemius	(a) no treatment, (b) deep tissue foam rolling, and (c) dynamic stretching FR protocol: 1 min on each muscle and extremity; 8 min in total	3 (1 per condition)
Murray, 2016 [75]	RCD	12 m (14.2 ± 1.4)	Elite squash sports academy	Hip flexors and quadriceps	FR and FAT groups (10 subjects each) FAT is a new form of IASTM For 60 s per muscle group, with 30 back-and-forth FR movements (15 in each direction)	2 (1 per condition)
Skarabot, 2015 [76]	RCD	11 (5 w), (6 m); (15.3 ± 1.0)	Trained endurance swimmers	Soleus, gastrocnemius	SS and FR groups: Each comprises three sets of 30 s of the intervention with 10 s of inter-set rest. FR + SS comprises the protocol from the FR condition followed by the protocol from the SS condition in sequence	3 (1 per condition)
Markovic, 2015 [77]	RCT	20 m (19 ± 2)	Competitive soccer	Quadriceps and hamstrings	FR group; FAT group (a new form of IASTM) Two sets × 1 min of FR per muscle group	2 (1 per condition)
Peacock, 2014 [78]	RCD	11 m (22.18 ± 2.18)	NCAA DI and DII competitive American football, soccer, track and field athletes	Thoracolumbar region, glutes, hamstrings, gastrocnemius, quadriceps, pectoral	The two warm-up routines compared: a total-body dynamic warm-up (DYN) and a total-body dynamic warm-up paired with SMR FR protocol: Five back-and-forth FR movements on each muscle group, taking 30 s × each back-and-forth movement; applied on both sides	2 (1 per condition)

CG = control group; DS = dynamic stretching; DYN = dynamic warm-up; FAT = fascial abrasion technique; FP = field player; FR or FRG= foam roller group; GK = goalkeeper; GW = general warm-up; IASTM = instrument-assisted soft-tissue mobilisation; m = men; min = minutes; MMA = mixed martial arts; NCAA DI/DII = National Collegiate Athletic Association I division/II division; PVPD = portable vibrational percussion devices; RCD = randomised crossover design; RCT = randomised controlled trial; Reps = repetitions; ROM = range of movement; s = seconds; SH = superficial heating; SMR= self-myofascial release; SI-SMR = short and intensive self-myofascial release; SS = static stretching; VR = vibration foam rolling; w = women.

**Table 3:** Effects of SMR on athletes’ flexibility and mobility.

Study	*n*	Measurement	Results			
			Group	M	SD	*p*
Kurt, 2023 [54]	23	Sit-and-reach test (cm)	SS	36.4	5.7	*p =* 0.01 * (SS-DS); *p* = 0.001 * (DS-SMR)
			DS	38.3	6.2	
			SMR	36.9	5.9	
Chen, 2023 [56]	10	Knee flexion (degrees)	GW	69.3	9.6	*p* < 0.05 *
			DS + VFR	79.4	7.7	
		Knee extension (degrees)	GW	130.5	6	*p* > 0.05
			DS + VFR	133.5	5.1	
Wang, 2022 [59]	27	Y balance test—Left	VFR	0.879	0.081	*p* > 0.05
			Percussion devices	0.849	0.074	
			CG	0.872	0.036	
		Y balance test—Right	VFR	0.876	0.123	
			Percussion devices	0.867	0.085	
			CG	0.878	0.064	
Chen, 2021 [60]	15	Active knee flexion—Stronger leg (degrees)	GW	127.9	5.3	*p* = 0.87
			GW + VFR	128.9	5.3	
		Active knee flexion—Weaker leg (degrees)	GW	129.1	4.9	*p* = 0.70
			GW + VFR	128.7	4.9	
		Modified sit-and-reach test—Stronger leg (degrees)	GW	59.8	9.9	*p* = 0.92
			GW + VFR	60.6	8.4	
		Modified sit-and-reach test—Weaker leg (degrees)	GW	57.1	11.1	*p* = 0.8
			GW + VFR	59.6	8.8	
Lopez Samanes, 2021 [61]	11	Passive straight leg raise test—Dominant leg (degrees)	DS pre	76.55	6.07	*p* > 0.05
			DS post	78.18	6.23	
			SMR pre	77.27	5.75	
			SMR post	79.09	4.13	
		Passive straight leg raise test—No dominant leg (degrees)	DS pre	75.64	5.78	*p* > 0.05
			DS post	76.36	5.71	
			SMR pre	77.09	5.47	
			SMR post	78.36	4.97	
		Modified version of the Thomas test—Dominant leg	DS pre	0.91	3.39	*p* > 0.05
			DS post	1.82	3.52	
			SMR pre	0	2.69	
			SMR post	0.36	2.8	
		Modified version of the Thomas test—No dominant leg	DS pre	2.18	3.16	*p* > 0.05
			DS post	2.55	3.24	
			SMR pre	0.73	2.24	
			SMR post	1.09	3.02	
Oranchuk, 2019 [63]	13	Passive straight leg raise (degrees)	CG	+1.9°		*p* > 0.05
			FR	+5.4°		*p* < 0.001 *
			FR + heat	+9.5°		*p* < 0.001 *
Romero-Franco, 2019 [64]	30	Thomas hip extension (degrees)	CG	−3.3–4.1°	Intra *p* < 0.05 *	Inter *p* > 0.05
			FR	−4.7–5.5°	Intra *p* < 0.05 *	
		Thomas knee flexion (degrees)	CG	+1.6°	Intra *p* > 0.05	Inter *p* < 0.05 *
			FR	−2.4°	Intra *p* < 0.05 *	
		Popliteal angle test (degrees)	CG	+1.8°	*p* > 0.05 intra and inter group	
			FR	−5.6°		
		Ankle dorsiflexion (degrees)	CG	+1.5°	Intra *p* > 0.05	Inter *p* > 0.05
			FR	+6.3°	Intra *p* < 0.05 *	
Guillot, 2019 [65]	30	Side split (degrees)	CG	+1.8°		*p* = 0.67
			FR20	+17.7°		*p* = 0.002 ^*^
			FR40	+18°		*p* = 0.005 ^*^
		Active straight leg raise—Right side (degrees)	CG	+0.6°		*p* = 0.84
			FR20	+14°		*p* = 0.004 *
			FR40	+6.2°		*p* = 0.002 *
		Active straight leg raise—Left side (degrees)	CG	+0.1°		*p* = 0.98
			FR20	+9.2°		*p* = 0.060
			FR40	+15.7°		*p* = 0.003 *
		Active flexed leg raise—Right side (degrees)	CG	+1.8°		*p* = 0.73
			FR20	+14.2°		*p* = 0.004 *
			FR40	+16.9°		*p* = 0.001 *
		Active flexed leg raise—Left side (degrees)	CG	−0.1°		*p* = 0.98
			FR20	+11.5°		*p* = 0.01 *
			FR40	+16.4°		*p* < 0.001 *
		Hip extension—Right side (degrees)	CG	+0.9		*p* = 0.76
			FR20	+17.1°		*p* < 0.001 *
			FR40	+15.4°		*p* < 0.001 ^*^
		Hip extension—Left side (degrees)	CG	+0.7		*p* = 0.82
			FR20	+15.5°		*p* < 0.001 *
			FR40	+13.5°		*p* < 0.001 ^*^
Aune, 2018 [66]	23	Dorsiflexion ROM (degrees)	Eccentric	+7%		*p* < 0.001 * pre–post (whole sample) Inter *p* > 0.05
			FR	+9%		
Richman, 2018 [69]	14	Sit and reach/flexibility (cm)	Light walking + DS pre	37.6	4.2	*p* < 0.05 *
			Light walking + DS post	39.9	3.9	
			FR + DS pre	36.3	4.8	*p* < 0.05 *
			FR + DS post	38.5	4.4	
Rey, 2017 [73]	18	Sit and reach (cm)	CG pre	25.27	8.8	*p* > 0.05
			CG post	24.94	7.24	
			FR pre	20.79	9.18	
			FR post	23.17	7.61	
Fairall, 2017 [72]	12	Glenohumeral internal rotation ROM (degrees)	SMR	24.15	4.66	Intra-group *p* < 0.000 * Inter-group *p* = 0.55
			SS	28.62	6.79	
			SMR + SS	30.36	7.21	
D’Amico, 2017 [71]	16	ROM hip extension (degrees)	Passive	Not specified		*p* > 0.05
			FR			
		Stride length (cm)	Passive	Not specified		*p* > 0.05
			FR			
Sağiroğlu, 2017 [70]	16	Sit-and-reach (cm) peak improvement during recovery (time)	Aerobic running	MIP = +2.13 cm (min 30:30)		Inter-group *p* < 0.05 * (aerobic running + SMR with better results in short term <10 min)
			Aerobic running + SS	MIP = +1.69 cm (min 15:30)		
			Aerobic running + SMR	MIP = +2.03 cm (min 10:30)		
Behara, 2017 [74]		ROM hip flexion (degrees)	Baseline	94.17	21.1	*p* < 0.000 *
			FR	108.92	15.51	
			DS	111.77	13.44	
Murray, 2016 [75]	12	ROM hip flexors and quadriceps (degrees)	FR + 2.4° than CG for overall flexibility changes			*p* = 0.03 *
Markovic, 2015 [77]	20	Passive straight leg raise (degrees)	FAT	+13.7° hip		*p* = 0.039 *
			FR	+6.6° hip		
		Passive knee flexion test (degrees)	FAT	+15.2°		*p* = 0.06
			FR	+7°		
Škarabot, 2015 [76]	11	ROM ankle dorsiflexion (degrees)	SS	+0.9	0.67	*p* < 0.05 *
			FR	+0.4	0.67	
			SS + FR	+1.3	0.65	
Peacock, 2014 [78]	11	Sit and reach (cm)	DYN	34.12	5.21	*p* = 0.83
			SMR + DYN	34.32	5.7	

CG = control group; cm = centimetres; DS = dynamic stretching; DYN = dynamic warm-up; FAT = fascial abrasion technique; FR = foam roller; GW = general warm-up; M = mean; MIP = maximal improvement; *p* ≤ 0.05 or * = significant; post = post-intervention; pre = pre-intervention; ROM = range of motion; SD = standard deviation; SMR = self-myofascial release; SS = static stretching; VFR = vibration foam roller.

**Table 4:** Effects of SMR on athletes’ strength.

Study	*n*	Measurement	Results			
			Group	M	SD	*p*
Kurt, 2023 [54]	23	CMJ height (cm)	SS	22.3	3	*p* < 0.05 *
			DS	25	3.7	
			SMR	23.8	0	
		CMJ reactive index	SS	0.528	2.275	*p* > 0.05
			DS	0.50	0.27	
			SMR	0.57	0.33	
		Stiffness (kN/m)	SS	6.92	6.4	*p* > 0.05
			DS	6.50	6.81	
			SMR	7.07	7.1	
		Isokinetic peak torque—right knee extensor at 60° (Nm)	SS	132.4	28.9	*p* = 0.038 *
			DS	140.4	25.6	
			SMR	138	24.6	
		Isokinetic peak torque—left knee extensor at 60° (Nm)	SS	125	24	*p* = 0.086
			DS	130.7	21.9	
			SMR	131.9	22.7	
		Isokinetic peak torque—right knee flexor at 60° (Nm)	SS	90	15.5	*p* = 0.006 *
			DS	94.9	18.5	
			SMR	94.7	17.1	
		Isokinetic peak torque—left knee flexor at 60° (Nm)	SS	88.3	13.5	*p* = 0.603
			DS	89.7	12.9	
			SMR	91	15	
Barrenetxea-García, 2023 [55]	30	In-water boost (jump, cm)	CG	116.04	6.82	*p* > 0.05
			FR	114.51	6.96	
		Throwing speed test (m.s^−1^)	CG	56.97	7	*p* > 0.05
			FR	58.08	7.88	
Chen, 2023 [56]	10	Hamstring stiffness (Nm^−1^)	GW	292.89	24.28	*p* = 0.01 *
			DS + VFR	253.33	36.2	
		Quadricep stiffness (Nm^−1^)	GW	254	23.78	*p* > 0.05
			DS + VFR	257.09	12.5	
		Hamstring isokinetic strength at 60° (Nm)	GW	51.52	8.89	*p* > 0.05
			DS + VFR	51.39	12.28	
		Quadricep isokinetic strength at 60° (Nm)	GW	107.47	14.29	*p* > 0.05
			DS + VFR	100.16	21.76	
Wang, 2022 [59]	27	CMJ (cm)	VFR	53.18	4.49	*p* = 0.03 *
			Percussion devices	50.08	3.97	
			CG	47.92	3.82	
		Drop jump (reactive strength index)	VFR	2.01	0.11	*p* = 0.012 *
			Percussion devices	1.99	0.11	
			CG	1.86	0.05	
Kozlenia, 2022 [57]	30	SJ (Jump height, relative force and power)	A vs. B	No SI-SMR vs. SI-SMR Diff.= 0.5–1.70 (A) vs. 0.11–0.16 (B) (depending on the variable)		*p* > 0.05
		CMJ (Jump height, relative force and power)	A vs. B	Diff. = 0.23–0.89 (A) vs. −0.18–0.41 (B) (depending on the variable)		*p* > 0.05
		CMJ Height (Eccentric utilisation ratio)	A vs. B	Diff.= 0.01 (A) vs. 0.03 (B)		*p* > 0.05
		DJ (Jump height, relative force and power, reactive strength index and stiffness)	A vs. B	Diff.= −0.03–3.31 (A) vs. −1.71–0.46 (B) (depending on the variable)		*p* > 0.05
Chen, 2021 [60]	15	CMJ (cm)	GW	34.6	4.1	*p* = 0.61
			GW + VFR	35.1	4.7	
Lopez Samanes, 2021 [61]	11	CMJ (cm)	DS pre	34.78	5.13	*p* > 0.05
			DS post	35.59	5.19	
			SMR pre	34.24	6.77	
			SMR post	34.42	7.07	
Rahimi, 2020 [62]	17	Sergeant jump (cm)	Passive pre	44.9	7	*p* > 0.05
			Passive post	43.8	6.7	
			FR pre	45.4	3	
			FR post	44.5	4.5	
Romero-Franco, 2019 [64]	30	CMJ (cm)	CG	+1.9	Intra *p* < 0.05 *	Inter *p* > 0.05
			FR	+4	Intra *p* < 0.05 *	
Aune, 2018 [66]	23	Drop jump—Reactive strength index	Significant chronic increase through the intervention (whole group, FR + eccentric)			Inter *p* = 0.932
		Plantar flexion torque (Nm)	Slightly better acute response for eccentric group, but no significant			*p* = 0.402
Richman, 2019 [69]	14	Drop jump (cm)	Light walk + DS	42.45	6.35	*p* = 0.351
			FR + DS	43.18	7.01	
		Squat jump (cm)	Light walk + DS	36.01	8.16	*p* = 0.022 *
			FR + DS	37.73	7.75	
		CMJ (cm)	Light walk + DS	40.91	7.66	*p* = 0.021 *
			FR + DS	43.54	7.26	
Stroiney, 2018 [68]	49	Sergeant jump—Men (cm)	SMR pre	59.41	6.48	*p* inter-group < 0.05 *
			SMR post	61.95	9.68	
			Assisted soft tissue mobilisation, pre	59.15	13.36	
			Assisted soft tissue mobilisation, post	60.22	11.07	
		Sergeant jump—Women (cm)	SMR pre	45.11	4.78	
			SMR post	46.3	6.68	
			Assisted soft tissue mobilisation, pre	47.55	7.26	
			Assisted soft tissue mobilisation, post	43.26	8.1	
Giovanelli, 2018 [67]	13	Maximal power—Lower limbs (W/kg)	CG	62.1	11.1	*p* = 0.251
			FR	58.9	15.7	
		CMJ/RFD (N)	FR pre	1819	362	*p* = 0.024 *
			FR post	1972	461	
Rey, 2017 [73]	18	CMJ (cm)	CG pre	32.33	5.43	*p* > 0.05
			CG post	30.36	4.53	
			FR pre	31.32	4.28	
			FR post	30.26	3.34	
Sağiroğlu, 2017 [70]	16	CMJ (cm) peak loss during recovery (time)	Aerobic running	−1.69 (min 30:00)		*p* inter-group > 0.05
			Aerobic running + SS	−2.62 (min 30:00)		
			Aerobic running + SMR	−2.19 (min 30:30)		
Behara, 2017 [74]	14	Vertical jump—Power peak (Watts)	Baseline	4282.91	487.81	*p* = 0.45
			FR	4372.46	474.57	
			DS	4318.73	418.52	
		Vertical jump—Velocity peak (m.s^−1^)	Baseline	3.18	0.32	*p* = 0.25
			FR	3.27	0.28	
			DS	3.22	0.27	
		Leg extension isometric force (Nm)	Baseline	221.63	40.15	*p* = 0.63
			FR	214.01	49.85	
			DS	208.44	60.25	
		Leg flexion isometric force (Nm)	Baseline	134	25.06	*p* = 0.63
			FR	125.13	17.53	
			DS	126.11	21.83	
Peacock, 2014 [78]	11	Vertical jump (cm)	DYN	67.66	9.79	*p* = 0.012 *
			SMR + DYN	72.97	10.6	
		Horizontal jump (cm)	DYN	228.6	25.25	*p* = 0.007 *
			SMR + DYN	237.84	25.45	
		Indirect 1RM bench press (kg)	DYN	99.92	19.56	*p* = 0.024 *
			SMR + DYN	103.68	20.47	

CG = control group; cm = centimetres; CMJ = counter-movement jump; Diff. = differences; DJ = drop jump; DS = dynamic stretching; DYN = dynamic warm-up; FR = foam roller; GW = general warm-up; kg = kilograms; m = metres; M = mean; Nm = Newtons · metre; *p* ≤ 0.05 or * = significant; post = post-intervention; pre = pre-intervention; RFD = rate of force development; s = seconds; SD = standard deviation; SI-SMR = short and intensive self-myofascial eelease; SJ = squat jump; SS = static stretching; VFR = vibration foam roller; W = Watts.

**Table 5:** Effects of SMR on speed.

Study	*n*	Measurement	Results			
			Group	M	SD	*p*
Barrenetxea-García, 2023 [55]	30	20 m sprint swim test (seconds)	CG	12.23	0.75	*p* > 0.05
			FR	12.17	0.89	
Wang, 2022 [59]	27	2.5 m lateral acceleration test left (seconds)	VFR	0.94	0.098	*p* > 0.05
			Percussion devices	1.004	0.138	
			CG	0.951	0.09	
		2.5 m lateral acceleration test (seconds)	VFR	0.896	0.1	
			Percussion devices	0.967	0.107	
			CG	0.954	0.122	
Lopez Samanes, 2021 [61]	11	10 m sprints (seconds)	DS pre	2.22	0.11	*p* > 0.05
			DS post	2.2	0.12	
			SMR pre	2.12	0.08	
			SMR post	2.14	0.08	
Rahimi, 2020 [62]	17	Repeated sprint ability (m)	Passive pre	740.8	52.3	*p* > 0.05
			Passive post	727.6	65.3	
			FR pre	723.5	41.9	
			FR post	689.6	40.8	
Stroiney, 2018 [68]		Sprint time—Men (seconds)	SMR pre	5.4	0.47	*p* > 0.05
			SMR post	5.47	0.52	
			Assisted soft tissue mobilisation, pre	5.1	0.42	
			Assisted soft tissue mobilisation, post	5.34	0.43	
		Sprint time—Women (seconds)	SMR pre	5.7	0.27	
			SMR post	6.06	0.5	
			Assisted soft tissue mobilisation, pre	5.8	0.49	
			Assisted soft tissue mobilisation, post	5.94	0.47	
Giovanelli, 2018 [67]	13	Running energy cost	CG pre vs. post	Not specified		
			FR pre vs. post	+6.2%	8.3	*p* = 0.052
D’Amico, 2017 [71]	16	Running time (seconds)	Active recovery	146.9	2.2	*p* < 0.05 *
			SMR	145.2	1.8	
Rey, 2017 [73]	18	5 m sprints (seconds)	CG pre	0.98	0.03	*p* > 0.05
			CG post	1	0.05	
			FR pre	0.98	0.06	
			FR post	1	0.06	
		10 m sprints (seconds)	CG pre	1.68	0.07	*p* > 0.05
			CG post	1.71	0.07	
			FR pre	1.71	0.09	
			FR post	1.72	0.05	
Peacock, 2014 [78]	11	37 m sprints (seconds)	DYN	5.11	0.29	*p* = 0.002 *
			SMR + DYN	4.95	0.21	

CG = control group; DS = dynamic stretching; DYN = dynamic warm-up; FR = foam roller; M = mean; *p* ≤ 0.05 or * = significant; post = post-intervention; pre = pre-intervention; SD = standard deviation; SMR = self-myofascial release; VFR = vibration foam roller.

**Table 6:** Effects of SMR on athletes’ agility.

Study	*n*	Measurement	Results			
			Group	M	SD	*p*
Wang, 2022 [59]	27	Hexagon test (seconds)	VFR	10.73	0.4	*p* = 0.03 *
			Percussion devices	11.02	0.45	
			CG	11.39	0.73	
Chen, 2021 [60]	15	Frequency speed of kick test	GW	105.1	7.7	*p* = 0.33
			GW + VFR	109.5	9.9	
		Hexagon test (seconds)	GW	12.8	1.6	*p* = 0.03 *
			GW + VFR	11.6	1	
		5-0-5 test (seconds)	GW	2.6	0.2	*p* = 0.37
			GW + VFR	2.6	0.2	
Lopez Samanes, 2021 [61]	11	5-0-5 test (seconds)	DS pre	2.86	0.14	*p* > 0.05
			DS post	2.79	0.13	
			SMR pre	2.75	0.18	
			SMR post	2.76	0.13	
Rahimi, 2020 [62]	17	Pro-agility 5-10-5 test (seconds)	Passive pre	5.2	0.3	*p* > 0.05
			Passive post	5.4	0.3	
			FR pre	5.1	0.3	
			FR post	5.2	0.3	
Richman, 2018 [69]	14	Short sprint (seconds)	Light walk + DS	2.05	0.17	*p* = 0.222
			FR + DS	2.02	0.13	
		*t*-test (seconds)	Light walk + DS	12.22	0.77	*p* = 0.577
			FR + DS	12.18	0.08	
Rey, 2017 [73]	18	*t*-test (seconds)	CG pre	9.22	0.21	*p* between groups < 0.05 *
			CG post	9.48	0.27	
			FR pre	9.34	0.31	
			FR post	9.36	0.34	
Peacock, 2014 [78]	11	Pro-agility 18.3 m (seconds)	DYN	4.97	0.24	*p* = 0.001 *
			SMR + DYN	4.8	0.16	

CG = control group; DS = dynamic stretching; DYN = dynamic warm-up; FR = foam roller; GW = general warm-up; m = metres; M = mean; *p* ≤ 0.05 or * = significant; post = post-intervention; pre = pre-intervention; SD = standard deviation; SMR = self-myofascial release; VFR = vibration foam roller.

**Table 7:** SMR effects on the recovery of athletes.

Study	*n*	Measurement	Results				
			Group	M		SD	*p*
Barrenetxea-García, 2023 [55]	30	sRPE	CG	min 580 max 750	145–181 res.		*p* > 0.05
			FR	min 627 max 829	248–150 res.		
Michalski, 2022 [58]	40	%MVC GM	HR	22.9 p0; 21.1 p1; 22.7 p2			*p* < 0.001 (p1)
			CG	38.9 p0; 4.2 p1; 36.1 p2			
		%MVC BF	HR	21.7 p0; 20.7 p1; 23 p2			*p* < 0.001 (p1); *p* < 0.01 (p2)
			CG	27 p0; 41.8 p1; 40.2 p2			
		sEMG GM	HR	109.8 p0; 89.7 p1; 100 p2			*p* < 0.001 (p1)
			CG	143 p0; 153.7 p1; 131.3 p2			
		sEMG BF	HR	114.8 p0; 109.9 p1; 121.3 p2			*p* < 0.0001 (p1); *p* < 0.001 (p2)
			CG	113 p0; 237.7 p1; 228.4 p2			
Chen, 2021 [60]	15	Rate of perceived exertion	FR	12.17		0.89	*p* = 0.93
			GW + VFR	6.7		1.6	
Rahimi, 2020 [62]	17	Hooper questionnaire	FR vs. PR Lower scores FR (2–3° md) FR vs. PR No difference FR vs. PR Differences at 3° md 4–6 vs. 5–8				*p* < 0.05 *
		Rate of perceived exertion					*p* > 0.05
		Blood lactate (mmol/L)					*p* < 0.05 *
Giovanelli, 2018 [67]	13	Rate of perceived exertion	CG pre	2.7		1.2	*p* = 0.586
			CG post	2.8		1.1	
			FR pre	2.6		1.1	*p* = 0.054
			FR post	2.2		0.9	
Rey, 2017 [73]	18	Total quality recovery test	CG pre	15.57		1.33	*p* between groups < 0.05 *
			CG post	12.67		1.66	
			FR pre	15.11		1.54	
			FR post	15.00		1.67	
		Visual analogue scale	CG pre	4.05		0.06	*p* between groups < 0.05 *
			CG post	5.6		1.19	
			FR pre	4.81		0.85	
			FR post	4.83		1.02	
D’Amico, 2017 [71]	16	Blood lactate (mmol/L)	Passive	Not specified			*p* > 0.05
			FR				
Murray, 2016 [75]	12	Tensiomyography	CG	No differences between protocols on any variables			*p* > 0.05
			FR				

BF = biceps femoris; CG = control group; sEMG = surface electromyography; FR = foam roller; GM = gluteus maximus; GW = general warm-up; HR = hamstring rolling; max = maximum; MVC = maximum voluntary contraction; md = match day; min = minimum; mmol/L = millimoles per litre; *p* ≤ 0.05 or * = significant; p0 = before rolling; p1 = immediately after; p2 = follow-up 5 min after p1; post = post-intervention; PR = passive rest; pre = pre-intervention; res. = respectively; sRPE = rate of perceived exertion within the session; VFR = vibration foam roller.

## Data Availability

There are no additional data beyond those provided in this article.
